# In Vitro Signaling Properties of Cannabinoid and Orexin Receptors: How Orexin Receptors Influence Cannabinoid Receptor‐Mediated Signaling

**DOI:** 10.1002/prp2.70078

**Published:** 2025-02-25

**Authors:** Kawthar A. Mohamed, Robert B. Laprairie

**Affiliations:** ^1^ College of Pharmacy and Nutrition University of Saskatchewan Saskatoon Saskatchewan Canada; ^2^ Department of Pharmacology, Faculty of Medicine Dalhousie University Halifax Nova Scotia Canada

**Keywords:** cAMP, cannabinoid receptors, cell signaling, orexin receptors, βarrestin2

## Abstract

The co‐expression of different types of G protein‐coupled receptors (GPCRs) in the same cells can have implications for receptor signaling and receptor cross‐talk, potentially altering the apparent potency or efficacy of ligands targeting each receptor. The endocannabinoid and orexinergic systems, consisting of class A GPCRs and their endogenous ligands, are highly complex and regulate processes such as appetite, sleep, nociception, and energy homeostasis. The shared anatomical distribution of cannabinoid and orexin receptors in various regions of the central nervous system (CNS), coupled with data from previous studies exploring physical and functional interactions between these receptors, suggests that the endocannabinoid and orexinergic systems engage in crosstalk. In this study, we explored how orexin receptors (OX_1_, OX_2_) altered the in vitro signaling of cannabinoid receptors (CB_1_, CB_2_) in Chinese hamster ovary (CHO)‐K1 cells by quantifying cyclic adenosine monophosphate (cAMP) inhibition and βarrestin2 recruitment. Our results suggest that orexin receptors alter agonist‐dependent signaling at the cannabinoid receptors by enhancing cannabinoid receptor‐mediated cAMP inhibition while increasing or decreasing cannabinoid receptor‐mediated βarrestin2 recruitment. These initial results are important for understanding the effects associated with cannabinoid ligands and may provide novel insights for therapeutics targeting physiological processes modulated by both systems.

## Graphical abstract

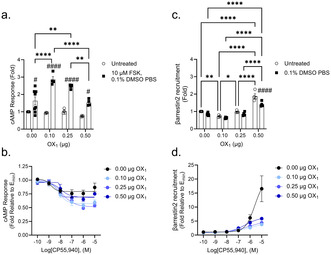

**Abbreviations**
2‐AG: 2‐arachidonoylglycerol
ANOVA: analysis of variance
BRET: bioluminescence resonance energy transfer
CB_1_
: type 1 cannabinoid receptor
CB_2_
: type 2 cannabinoid receptor
CHO: Chinese hamster ovary
CI: confidence interval
CNS: central nervous system
CRC: concentration‐response curve
DAG: diacylglycerol
DAGL: diacylglycerol lipase
DMEM: Dulbecco's Modified Eagle Medium
DMSO: dimethyl sulfoxide
DORA: dual orexin receptor antagonist
EA: enzyme acceptor
ECS: endocannabinoid system
ED: enzyme donor
EFC: enzyme fragment complementation
eGFP: enhanced green fluorescent protein
FBS: fetal bovine serum
FRET: fluorescence resonance energy transfer
FSK: forskolin
GPCR: G protein‐coupled receptor
IP3: inositol trisphosphate
MAPK: mitogen‐activated protein kinase
n.c.: not converged
OX_1_
: orexin receptor type 1
OX_2_
: orexin receptor type 2
OXA: orexin A
OXB: orexin B
PBS: phosphate‐buffered saline
Pen/Strep: penicillin–streptomycin
PLC: phospholipase C

## 1 Introduction

G protein‐coupled receptors (GPCRs) play a critical role in cellular signaling, converting various external stimuli (e.g., photons, ions, odorants, hormones, and neurotransmitters) into intracellular responses [1, 2]. It has previously been demonstrated that GPCRs can interact with each other physically through receptor dimerization, a process that may involve two identical receptors (i.e., homodimers) or two distinct receptors (i.e., heterodimers) or indirectly via their convergent signal transduction pathways [3, 4, 5]. Ligand binding, receptor signaling, and receptor trafficking may be altered as a result of these GPCR interactions when receptors are co‐expressed in the same cells [6, 7].

The endocannabinoid system (ECS) and orexinergic system are highly complex biological systems that regulate key physiological processes. Within the ECS, endogenous cannabinoids (endocannabinoids) are synthesized “on demand” from cell membrane lipids and activate a pair of GPCRs, namely the type 1 and type 2 cannabinoid receptors (CB_1_ and CB_2_ , respectively) [8, 9]. Similarly, the orexinergic system includes orexins A and B (OXA and OXB), hypothalamic neuropeptides formed following proteolytic processing of the prepro‐orexin polypeptide [10]. These endogenous ligands activate the orexin receptor type 1 (OX_1_ ) and orexin receptor type 2 (OX_2_ ), a pair of GPCRs [11]. Cannabinoid and orexin receptors both belong to the rhodopsin‐like (class A) family of GPCRs [12]. However, CB_1_ and CB_2_ primarily couple to Gα_i/o_ G proteins, leading to reduced cAMP production via inhibition of adenylate cyclase, while OX_1_ and OX_2_ primarily couple to Gα_q_ G proteins, leading to the production of diacylglycerol (DAG) and inositol trisphosphate (IP_3_) via phospholipase C (PLC) activation [13, 14, 15].

The endocannabinoid and orexinergic systems are both implicated in the regulation of physiological processes such as appetite, sleep, reward, memory, nociception, inflammation, and energy homeostasis [12, 16]. This, coupled with the shared neuroanatomical distribution of CB_1_, OX_1_, and OX_2_ in the hypothalamus, mesocorticolimbic system, brainstem, and other regions of the central nervous system (CNS) suggests that crosstalk may exist between the endocannabinoid and orexinergic systems [12, 17]. Several studies have explored cellular and molecular interactions between CB_1_ and the orexin receptors. In cells expressing both CB_1_ and OX_1_, a CB_1_‐dependent mechanism is responsible for the 100‐fold increase in potency for OXA in the mitogen‐activated protein kinase (MAPK) signaling pathway via OX_1_ [18]. Furthermore, when CB_1_ and OX_1_ are co‐expressed, the potency of OXA or the synthetic cannabinoid WIN55,212‐2 to phosphorylate ERK1/2 is reduced when treated with the CB_1_ antagonist/inverse agonist SR141716 or the OX_1_ antagonist SB674042, respectively [19]. Additionally, OX_1_ activation by OXA has been shown to stimulate the synthesis of the endocannabinoid 2‐arachidonoylglycerol (2‐AG) via the Gα_q_ G protein‐mediated PLCβ‐diacylglycerol lipase‐α (DAGLα) pathway both in vitro and in vivo [20, 21]. This 2‐AG can subsequently activate CB_1_ in either an autocrine or paracrine manner [17, 21, 22]. OXA, which has no affinity for CB_1_, can also promote the internalization of CB_1_ but only when co‐expressed with OX_1_. Furthermore, OXA exhibits greater potency in promoting the internalization of CB_1_‐OX_1_ heterodimers over OX_1_ homomers [23]. Far less is known about CB_2_‐orexin receptor interactions, though CB_2_ and OX_1_ may form heterotetramers [24].

In this study, we investigated how orexin receptors can alter cannabinoid receptor‐mediated signaling in vitro. Chinese hamster ovary (CHO)‐K1 cells stably expressing CB_1_ or CB_2_ were transfected with OX_1_ or OX_2_ plasmids and evaluated in enzyme fragment complementation (EFC) assays quantifying cyclic adenosine monophosphate (cAMP) inhibition and βarrestin2 recruitment to explore downstream signaling at the cannabinoid receptors. Determining how cannabinoid receptor signaling is influenced by additional GPCRs, such as the orexin receptors, can improve our understanding of the effects of cannabinoid ligands and may uncover novel targets for therapeutic exploitation, especially for physiological processes modulated by both endocannabinoid and orexinergic systems.

## 2 Materials and Methods

### 2.1 Compounds

CP55,940 [(−)‐*cis*‐3‐[2‐Hydroxy‐4‐(1,1‐dimethylheptyl)phenyl]‐*trans*‐4‐(3‐hydroxypropyl)cyclohexanol] was purchased from Tocris Biosciences (Oakville, ON). Forskolin (FSK) was purchased from Sigma‐Aldrich (Mississauga, ON). CP55,940 and FSK were dissolved in dimethyl sulfoxide (DMSO) with a final concentration of 0.1% DMSO in PBS in assay media for all assays and added directly to media at the concentrations and times indicated.

### 2.2 Cell Culture

HitHunter (cAMP) and PathHunter (βarrestin2) CHO‐K1 cells stably expressing human CB_1_ or CB_2_ were obtained from DiscoverX (Eurofins, Fremont, CA, USA) and were maintained at 37°C and 5% CO_2_ in F‐12/Dulbecco's Modified Eagle Medium (DMEM) containing 1 mM L‐glutamine, 10% fetal bovine serum (FBS), and 1% penicillin–streptomycin (Pen/Strep). Cells were supplemented with 800 μg/mL geneticin (HitHunter) or 800 μg/mL geneticin and 300 μg/mL hygromycin B (PathHunter) [25, 26].

### 2.3 Plasmids

Human OX_1_‐enhanced green fluorescent protein (eGFP) and OX_2_‐eGFP plasmids (henceforth simply described as OX_1_ and OX_2_) were sourced from Proxima Research & Development (Saskatoon, SK, Canada). All plasmids were propagated and sequenced by Proxima Research & Development. For all plasmids, receptor cDNA was inserted into the common pcDNA3.1(+) vector. Receptor plasmids used in transfection experiments are described further in Supporting Information (Figure S1).

### 2.4 Transfections and Microscopy

Receptor plasmids were introduced into HitHunter CHO‐K1 cAMP cells and PathHunter CHO‐K1 βarrestin2 cells using Lipofectamine 3000, according to the manufacturer's instructions (Invitrogen, ThermoFisher Scientific, Waltham, MA, USA). For experiments where cAMP inhibition or βarrestin2 recruitment was measured, cells were grown to ~80% confluency in 10 cm tissue culture plates in F‐12/DMEM containing 1 mM L‐glutamine, 10% FBS, 1% Pen/Strep at 37°C and 5% CO_2_. Cell media was aspirated and cells were washed with 2 mL 1X phosphate‐buffered saline (PBS). Cells were then incubated in serum‐free media for 30 min at 37°C. Lipofectamine 3000 solution was prepared by diluting Lipofectamine 3000 reagent in Opti‐MEM. Plasmid solution was prepared by diluting plasmid DNA in Opti‐MEM and P3000 reagent. Diluted Lipofectamine 3000 reagent was then added to the diluted plasmid solution and the resultant solution was incubated for 15 min at room temperature. Following this, the Lipofectamine plasmid solution was added to the cells and cells were incubated for 24–48 h at 37°C and 5% CO_2_ prior to conducting experiments. HitHunter CHO‐K1 cAMP cells and PathHunter CHO‐K1 βarrestin2 cells were transfected with increasing amounts of OX_1_ or OX_2_ plasmid (0.10, 0.25, and 0.50 μg). CB_1_ or CB_2_ cells described in figures as receiving 0.00 μg OX_1_ or 0.00 μg OX_2_ (i.e., non‐transfected cells) were used as controls that received a ‘mock' transfection of only the Lipofectamine 3000 and P3000 reagent but without plasmid DNA and were otherwise identical to plasmid transfections. For microscopy experiments, cells were grown to ~50% confluency in 12‐well tissue culture plates in F‐12/DMEM containing 1 mM L‐glutamine, 10% FBS, and 1% Pen/Strep at 37°C and 5% CO_2_. Cell media was aspirated and cells were washed with 250 μL 1X PBS. Cells were then incubated in serum‐free media for 30 min at 37°C. Lipofectamine 3000 solution was prepared by diluting Lipofectamine 3000 reagent in Opti‐MEM. Plasmid solution was prepared by diluting plasmid DNA in Opti‐MEM and P3000 reagent. Diluted Lipofectamine 3000 reagent was then added to the diluted plasmid solution, and the resultant solution was incubated for 15 min at room temperature. Following this, the Lipofectamine plasmid solution was added to the cells, and cells were incubated for 48 h at 37°C and 5% CO_2_ prior to visualization. HitHunter CHO‐K1 cAMP cells and PathHunter CHO‐K1 βarrestin2 cells were transfected with 0.50 μg of OX_1_ or OX_2_ plasmid. CB_1_ or CB_2_ cells described in figures as receiving 0.00 μg OX_1_ or 0.00 μg OX_2_ (i.e., non‐transfected cells) were used as controls that received a ‘mock’ transfection of only the Lipofectamine 3000 and P3000 reagent but without plasmid DNA and were otherwise identical to plasmid transfections. Hoechst nuclei stain (100 mg, ThermoFisher Scientific) was dissolved in 10 mL *d*H_2_O to create a 10 mg/mL stock solution. The stock Hoechst stain was further diluted 1:5000 in PBS. Forty‐eight hours after transfection, cell media was removed and replaced with 250 μL of diluted Hoechst stain in PBS and incubated for 10 min at room temperature protected from light. The staining solution was then removed, and cells were washed three times in 250 μL PBS. After removing the last PBS, cells were provided with 200 μL FluoroBrite DMEM media (ThermoFisher Scientific). Cells were visualized using a ZOE fluorescent cell imaging system with a 20× objective using brightfield, blue fluorescent, and green fluorescent channels. Brightfield imaging settings were gain 29, exposure 220, LED intensity 26, and contrast 35. Blue (for nuclei) settings were gain 41, exposure 250, LED intensity 7, and contrast 38. Green (for OX receptor eGFP) settings were gain 109, exposure 260, LED intensity 56, and contrast 59. Images were exported as .tif files for subsequent analysis detailed in Section 2.7.

### 2.5 HitHunter cAMP Assay

Gα_i/o_‐mediated inhibition of cAMP production was assessed using the DiscoverX HitHunter assay in CHO‐K1 CB_1_ and CB_2_ cAMP cells. CHO‐K1 cells used in the HitHunter assay stably express unmodified human CB_1_ or CB_2_ along with an enzyme acceptor (EA) portion of a modified β‐galactoside enzyme. cAMP levels are quantified through an EFC approach that relies on a modified β‐galactoside reaction to produce a chemiluminescent signal. In the HitHunter assay, cells are first incubated with ligand—in our case the cannabinoid receptor agonist CP55,940—that may alter cAMP levels via GPCR‐dependent signal transduction through Gα_i/o_ proteins. After this incubation period, cells are treated with a cAMP working solution containing enzyme donor (ED)‐conjugated cAMP and an anti‐cAMP antibody solution. The ED‐conjugated cAMP competes with cellular cAMP for binding to the anti‐cAMP antibodies. The ED‐conjugated cAMP is free to complement the EA to form active β‐galactoside by EFC. Last, a modified β‐galactoside substrate is added that can be hydrolyzed by the whole enzyme to produce light. In this competition, the antibody‐bound ED‐cAMP cannot complement the EA, but unbound ED‐cAMP can complement EA to form active enzyme, consequently producing luminescence. The amount of signal produced is directly proportional to the amount of cAMP in the cells. In our assays measuring cAMP changes in response to GPCR signaling via a Gα_i/o_‐coupled receptor, cAMP levels (and therefore chemiluminescence) decline. From a technical standpoint, the assay proceeded as follows: cells were dissociated from 10 cm tissue culture plates using 2 mL 0.5% trypsin–EDTA and replated into white‐walled, white‐bottom, flat‐bottom 96‐well tissue culture plates at a density of 20,000 cells/well. Plates were incubated overnight in Opti‐MEM containing 1% FBS at 37°C and 5% CO_2_. The next day, Opti‐MEM media was removed and replaced with cell assay buffer (1X PBS). Subsequently, cells were left untreated or treated with 0.1% DMSO in PBS containing 10 μM FSK and 0.1 nM to 10 μM ligands for 90 min at 37°C. cAMP antibody solution and ED‐conjugated cAMP solution were then added to cells according to the manufacturer's directions (DiscoverX). Cells were incubated with these solutions for 60 min at room temperature protected from light. β‐galactoside substrate (i.e., cAMP solution A) was added according to the manufacturer's directions (DiscoverX) and cells were incubated for an additional 3 h at room temperature protected from light. Chemiluminescence was measured on a BioTek Synergy HT microplate reader (BioTek Instruments, Winooski, VT) with the following settings: top read, gain 200, integration time 1000 ms [25, 26]. Raw data for the untreated and vehicle with FSK‐treated controls is presented in Figure S2.

### 2.6 PathHunter βarrestin2 Assay

βarrestin2 recruitment was assessed using the DiscoverX PathHunter assay in CHO‐K1 CB_1_ and CB_2_ βarrestin2 cells. CHO‐K1 cells used in the PathHunter assay stably express human CB_1_ or CB_2_ conjugated to the ED portion of a modified β‐galactoside enzyme and βarrestin2 conjugated to the EA portion of a modified β‐galactoside enzyme. Recruitment of βarrestin2 to a GPCR is quantified through an EFC approach that relies on a modified β‐galactoside reaction to produce a chemiluminescent signal. In the PathHunter assay, cells are first incubated with ligand—in our case the cannabinoid receptor agonist CP55,940—that may result in the recruitment of βarrestin2 to the GPCR. After this incubation period, cells are treated with a modified β‐galactoside substrate that can be hydrolyzed by the whole enzyme to produce light. The amount of signal produced is directly proportional to the amount of βarrestin2 recruitment occurring in the cells. From a technical standpoint, the assay proceeded as follows: cells were dissociated from 10 cm tissue culture plates using 2 mL 0.5% trypsin–EDTA and replated into white‐walled, white‐bottom, flat‐bottom 96‐well tissue culture plates at a density of 20,000 cells/well. Plates were incubated overnight in Opti‐MEM containing 1% FBS at 37°C and 5% CO_2_. Afterwards, cells were left untreated or treated with 0.1% DMSO in PBS or 0.1 nM–10 μM ligands for 90 min at 37°C. Detection solution (i.e., β‐galactoside substrate) was then added to cells according to the manufacturer's directions (DiscoverX) and cells were incubated for 60 min at room temperature. Chemiluminescence was measured on a BioTek Synergy HT microplate reader (BioTek Instruments, Winooski, VT) with the following settings: top read, gain 200, integration time 1000 ms [25, 26].

### 2.7 Image Analysis

Image files were imported into Fiji ImageJ v. 1.54i [27] to count the number of OX_1_ or OX_2_‐expressing cells based on their green fluorescence as a percentage of the number of Hoechst‐stained cells. Imported .tif images were first duplicated and the duplicates converted from RGB to 8‐bit format; then, an image threshold adjustment was applied using the “Threshold” tool with default settings. Images were then processed into Binary images and converted to a Mask, again using default settings. The “Watershed” tool was then applied to separate clusters of cells that would otherwise be counted as single objects. Cells were then counted using the “Analyze Particles” tool with the particle size set to 10—infinity pixels^2^ and all other settings kept as default. The percentage of receptor‐expressing cells was then estimated by dividing the number of green cells in a given field of view by the number of Hoechst‐stained nuclei in that same field of view, multiplied by 100.

### 2.8 Statistical Analyses

Statistical analyses were conducted using GraphPad Prism 9.0 (GraphPad Software Inc., San Diego, CA, USA). All assays were conducted with a minimum *n* = 4 independent experiments as indicated in figure legends with 3 technical replicates per treatment per experiment. In the whole of the study, 3 technical replicate datapoints from a single experiment were excluded because the experimenter noted technical issues in the execution of that experiment. Data for cAMP inhibition and βarrestin2 recruitment in untreated cells and cells receiving 0.1% DMSO +10 μM FSK in PBS (cAMP) or 0.1% DMSO in PBS (βarrestin2) are represented as fold compared to non‐transfected untreated cells (i.e., 1; Figures 2A,C, 3A,C, 4A,C, and 5A,C) For clarity this meant that in cAMP experiments the vehicle treatment contained 0.1% DMSO +10 μM FSK in PBS; and for βarrestin2 experiments the vehicle contained 0.1% DMSO in PBS. For all concentration‐response curves (CRC; Figures 2B,D, 3B,D, 4B,D, and 5B,D), raw data were transformed in GraphPad Prism using the formula “Y = Y/K” where ‘K' represented the mean *E*_min_ observed for the CRC obtained from raw data in a single 96‐well plate for that treatment group. In considering our data, several analytical approaches were considered; however, because the level of receptor expression was not measured within the cells used for experiments we did not think it appropriate to normalize to a set compound‐based parameter such as % FSK response. Therefore, we provide the data for cellular responses to no treatment and the solutions of 0.1% DMSO +10 μM FSK in PBS (cAMP) or 0.1% DMSO in PBS (βarrestin2) alongside the concentration‐response data. CRCs were fit using variable slope non‐linear regression (i.e., 4‐parameters) or the Hill slope was constrained to 1, where specifically noted. CRCs were used to estimate pEC_50_ and *E*_max_ values. Statistical analyses were conducted by one‐way analysis of variance (ANOVA) or two‐way ANOVA, as indicated in the figure and table descriptions. *Post hoc* analyses were performed using Tukey's test. All values are expressed as the mean ± the standard error of the mean (SEM) or 95% confidence interval (CI), as indicated. *p* values < 0.05 were considered significant.

### 2.9 Nomenclature of Targets and Ligands

Key protein targets and ligands in this article are hyperlinked to corresponding entries in http://www.guidetopharmacology.org, the common portal for data from the IUPHAR/BPS Guide to PHARMACOLOGY [28], and are permanently archived in the Concise Guide to PHARMACOLOGY 2019/20 [29].

## 3 Results

### 3.1 Estimation of Transfection Efficiency

**FIGURE 1:**
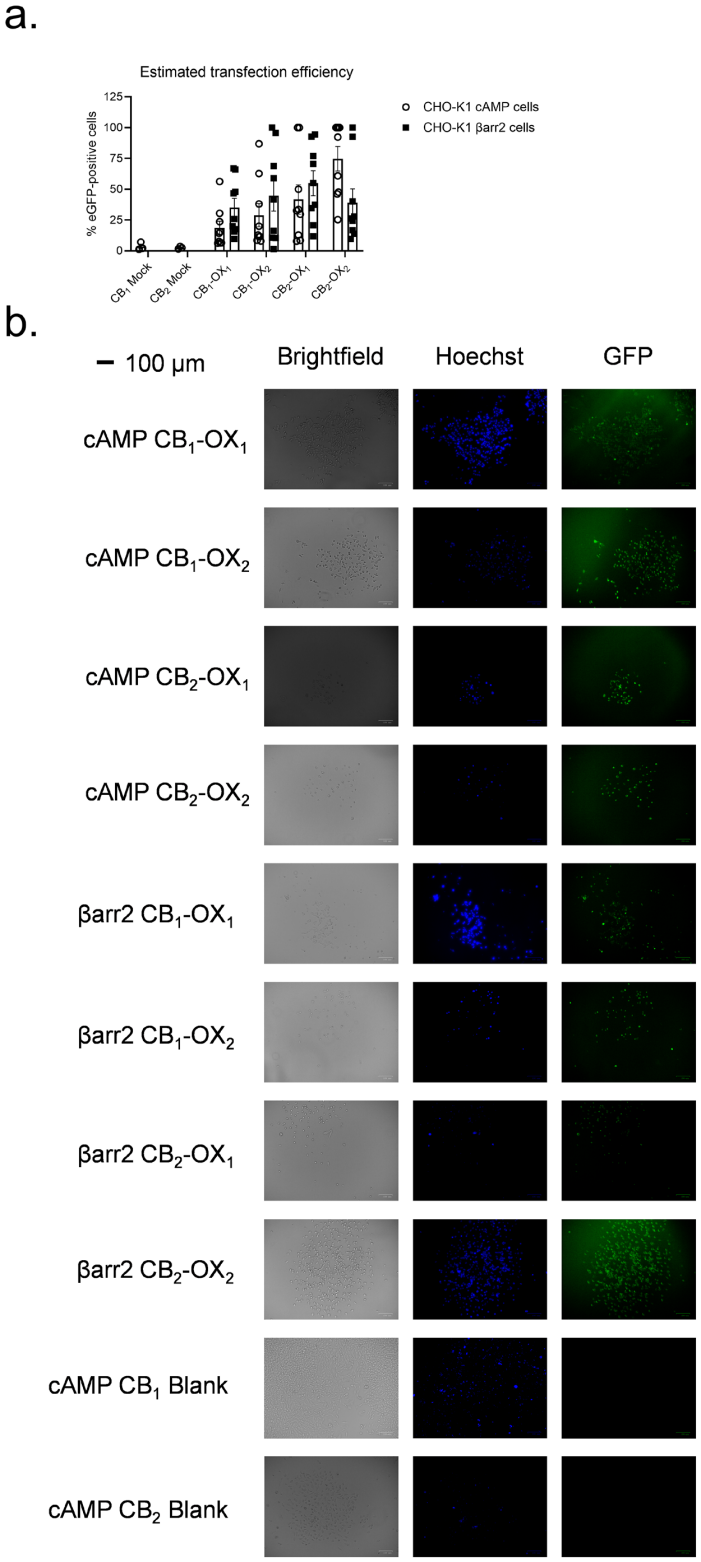
Estimation of transfection efficiency. CHO cells stably expressing CB_1_ and transiently transfected with either OX_1_ or OX_2_‐eGFP plasmids were stained with Hoechst to visualize nuclei and observed to estimate transfection efficiency based on the number of cells expressing eGFP (A). Data are mean ± SEM from *n* = 3 (Mock) or 9 independent experiments. (B) Representative images for data presented in panel (A). Scale bar represents 100 μm.

Because our experimental approach utilized CHO‐K1 cells stably expressing cannabinoid receptors but transiently transfected with orexin receptors, the transfection efficiency of Lipofectamine 3000 was estimated by counting the number of eGFP‐expressing cells relative to the number of Hoechst‐stained nuclei in a given field of view 48 h after transfection. The lowest transfection efficiency was observed in CHO‐K1 cAMP CB_1_ cells transfected with OX_1_ (19%) and the highest transfection efficiency was observed in CHO‐K1 cAMP CB_2_ cells transfected with OX_2_ (75%) (Figure 1A). However, transfection efficiency was not different between CB_1_‐expressing cells and CB_2_‐expressing cells. A subset of CHO‐K1 cAMP cells was also treated only with Lipofectamine 3000 for this experiment (i.e., ‘Mock’ transfected) and some green background fluorescence was observed in these cells using the same microscope and ImageJ analysis settings, such that the percentage of eGFP‐positive cells in these experiments was 3.5% and 2.6% for CB_1_ and CB_2_, respectively (Figure 1A). Representative micrographs are displayed in Figure 1B, and a full catalogue of all images is displayed in Supporting Information (Figure S3).

### 3.2 Modulation of cAMP and βarrestin2 Recruitment in CB_1_ ‐OX_1_ Transfected Cells

**FIGURE 2:**
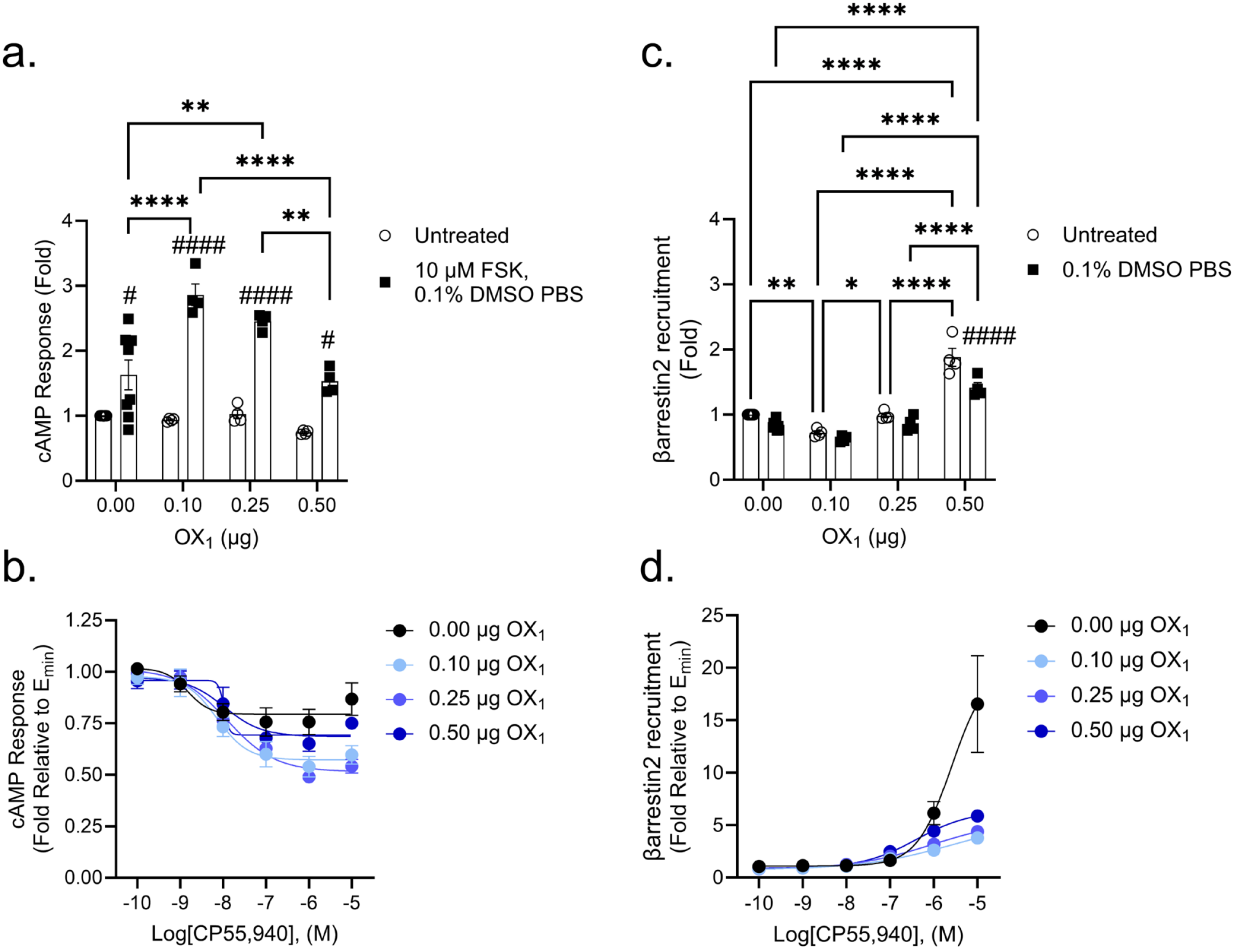
CB_1_‐dependent inhibition of cAMP and βarrestin2 recruitment in CB_1_‐OX_1_ cells. CHO cells stably expressing CB_1_ and transiently expressing OX_1_ were treated with 10 μM FSK and 0.1 nM—10 μM CP55,940 for 90 min to measure cAMP inhibition (A, B) or 0.1 nM—10 μM CP55,940 for 90 min to measure βarrestin2 recruitment (C, D). Data were fit to a 4‐parameter non‐linear regression and are represented as fold over vehicle. Data are mean ± SEM from *n* = 4–8 independent experiments performed in triplicate. *p < 0.05, **p < 0.01, ****p < 0.0001 within treatment between groups; ^#^ *p* < 0.05, ^####^ *p* < 0.0001 within groups between treatments as determined by two‐way ANOVA followed by Tukey's post hoc test.

In untreated cells, increasing the amount of OX_1_ did not significantly change the cAMP response (Figure 2A). Cells treated with the compound solvent, which included 0.1% DMSO and 10 μM FSK, saw statistically significantly higher levels of cAMP as expected following FSK treatment; and this effect was greater in cells transfected with 0.10 and 0.25 μg OX_1_ compared to non‐transfected cells or cells transfected with 0.50 μg OX_1_ (Figure 2A). Transfected and non‐transfected cells were treated with increasing concentrations of CP55,940 (Figure 2B). These data were first transformed relative to the *E*_min_ for each CRC as described in the Methods. Doing so constrained the CRC *E*_min_ to 1 so that curves for different amounts of transfected plasmid that had variable fold responses in Figure 2A were made comparable in Figure 2B. By constraining the *E*_min_ to 1 in this way, differences in potency and efficacy as a function of CP55,940 were compared under each transfection condition that were not overestimated through the inclusion of different basal vehicle conditions. In cells that were not transfected with OX_1_, a concentration‐response for CP55,940 was observed with a potency of 1.5 nM (Figure 2B); however, the goodness of fit was relatively low (*R* ^2^ = 0.25) albeit with reasonable residual standard deviation (Sy.x = 0.16) and so conclusions about shifts in potency should be interpreted with caution. Among transfected cells, as the amount of OX_1_ increased, CP55,940's potency decreased; however, this shift was not statistically significant (Figure 2B and Table 1). E_max_ was greater in cells transfected with 0.10 or 0.25 μg OX_1_, but cells transfected with 0.50 μg OX_1_ did not display greater efficacy compared to cells without OX_1_ (Figure 2B and Table 1). In addition, transfection with 0.50 μg OX_1_ resulted in a CRC that could not be fit to a 4‐parameter model (Table 1). This may indicate that at plasmid levels above 0.25 μg OX_1_, there may have been signaling interference as a result of OX_1_ coupling to Gα_i/o_ or Gα_S_ because coupling to these G proteins has been detected reviewed in [30]. Alternatively, OX_1_‐mediated signaling via Gα_q_ interfered with Gα_i/o_ signaling [30]. Overall, these data suggest that the presence of OX_1_ enhanced CB_1_‐dependent cAMP inhibition. In untreated cells, 0.10 μg OX_1_ reduced CB_1_‐dependent βarrestin2 recruitment; whereas 0.25 μg OX_1_ was not different from cells without OX_1_, and 0.50 μg OX_1_ produced greater βarrestin2 recruitment than 0.00, 0.10, or 0.25 μg OX_1_ in untreated cells (Figure 2C). Similarly, in cells that received 0.1% DMSO in PBS, βarrestin2 recruitment was greater in cells transfected with 0.50 μg OX_1_ compared to cells transfected with 0.00, 0.10, or 0.25 μg OX_1_ (Figure 2C). An effect of vehicle (0.1% DMSO in PBS) compared to untreated was present for cells transfected with 0.50 μg OX_1_; it is not clear whether this effect was biologically significant, but it is reported to show the vehicle did affect our observations under some conditions (Figure 2C). As with Figure 2A,B, CRC data in Figure 2D were constrained to an *E*_min_ of 1 to allow for comparisons of potency and efficacy following CP55,940 treatment. The CRC for CP55,940 in CHO‐K1 CB_1_ βarrestin2 cells that were not transfected did not reach a clear asymptote, and so the reported *E*_max_ is based on the observed result rather than the nonlinear regression (Figure 2D). In comparison, the potency of CP55,940 among transfected cells appeared to increase as the amount of OX_1_ increased, although this trend was not statistically significant (Table 1). The presence of OX_1_ reduced CP55,940's efficacy in the βarrestin2 recruitment assay, although this was not statistically significant (Figure 2D and Table 1). These data suggest that the effect of OX_1_ on CB_1_‐dependent βarrestin2 recruitment may depend on the specific treatment the cells are exposed to: βarrestin2 recruitment increased as a function of transfecting higher amounts of OX_1_ into cells while CP55,940‐treated cells exhibited reduced βarrestin2 recruitment.

**TABLE 1:** CB_1_‐dependent inhibition of cAMP and recruitment of βarrestin2 in CB_1_‐OX_1_ cells.

[OX_1_] (μg)	cAMP			βarrestin2		
	pEC_50_ (nM)	*E*_max_ (Fold)	Hill slope	pEC_50_ (nM)	*E*_max_ (Fold)^b^	Hill slope
0.00	8.8 ± 0.45 (1.5)	0.79 ± 0.03	−1.7 ± 3.6	< 5 (> 10 000)	17 ± 4.6	n.d.
0.10	8.1 ± 0.24 (7.2)	0.57 ± 0.03**	−1.2 ± 0.90	5.5 ± 1.4 (3000)	5.7 ± 3.2	0.41 ± 0.23
0.25	7.9 ± 0.16 (12)	0.52 ± 0.03***	−0.77 ± 0.22	6.0 ± 0.26 (960)	5.6 ± 0.63	0.46 ± 0.08
0.50	7.9 ± 0.33 (11)^a^	0.69 ± 0.03^a^	−1^a^	6.4 ± 0.068 (400)	6.4 ± 0.21	0.70 ± 0.06

*Note:* Data were fit to a 4‐parameter non‐linear regression to estimate potency (pEC_50_) and efficacy (*E*_max_) values. Data are expressed as mean ± SEM. *n* = 4–8 independent experiments performed in triplicate. Statistical analyses were by one‐way ANOVA followed by Tukey's post hoc test. Data presented in the Table relate to Figure 2.

Abbreviation: n.d., not determined.

** *p* < 0.01 relative to CP55,940.

*** *p* < 0.001 relative to CP55,940.

^a^ When fit to a 4‐parameter model, pEC_50_ and Hill Slope could not be accurately estimated, data in table are for a Hill Slope constrained to 1.

^b^ *E*_max_ data represent the experimentally observed value because CRC did not reach a clearly defined maximum asymptote; for this reason EC_50_ is reported as > 10 000 nM.

### 3.3 Modulation of cAMP and βarrestin2 Recruitment in CB_1_ ‐OX_2_ Transfected Cells

**FIGURE 3:**
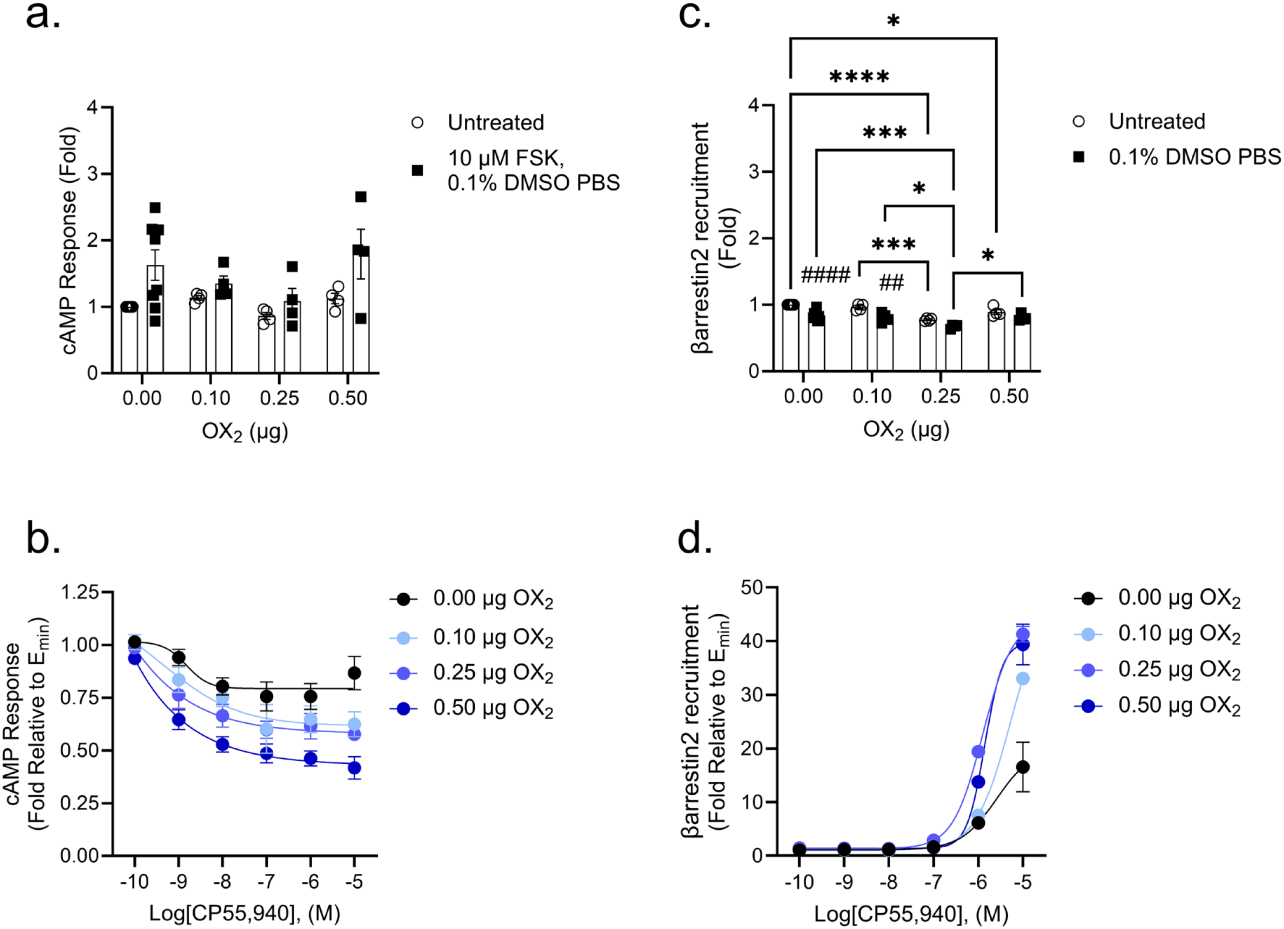
CB_1_‐dependent inhibition of cAMP and βarrestin2 recruitment in CB_1_‐OX_2_ cells. CHO cells stably expressing CB_1_ and transiently expressing OX_2_ were treated with 10 μM FSK and 0.1 nM–10 μM CP55,940 for 90 min to measure cAMP inhibition (A, B) or 0.1 nM–10 μM CP55,940 for 90 min to measure βarrestin2 recruitment (C, D). Data were fit to a 4‐parameter non‐linear regression and are represented as fold over vehicle. Data are mean ± SEM from *n* = 4–8 independent experiments performed in triplicate. *p < 0.05, ***p < 0.001, ****p < 0.0001 within treatment between groups; ^##^ *p* < 0.01, ^####^ *p* < 0.0001 within groups between treatments as determined by two‐way ANOVA followed by Tukey's post hoc test.

In untreated cells, no significant difference was observed in the cAMP response as the amount of OX_2_ increased (Figure 3A). In cells receiving 0.1% DMSO in PBS with 10 μM FSK, there was a trend toward increased cAMP levels with FSK, but these differences were not significant; similar to untreated cells, OX_2_ transfection did not yield a significant difference in cAMP response (Figure 3A). Data for CB_1_‐expressing cells that were not transfected with OX_1_ are identical in Figures 2B and 3B; therefore, because the goodness of fit was relatively low (*R* ^2^ = 0.25) albeit with reasonable residual standard deviation (Sy.x = 0.16) conclusions about shifts in potency should be interpreted with caution. As in Figure 2, CRC data in Figure 3B,D were constrained such that *E*_min_ = 1 to allow for comparisons of potency and efficacy following CP55,940 treatment. Among transfected cells, the presence of OX_2_ enhanced both the potency and efficacy of CP55,940, although the shift in potency was not assessed by ANOVA because the estimated potency was outside the range of CP55,940 used in the assay (i.e., < 0.1 nM) (Figure 3B and Table 2). These data suggest that OX_2_ enhanced CP55,940‐mediated cAMP inhibition via CB_1_. In untreated cells and cells that received 0.1% DMSO in PBS, transfection with 0.25 or 0.50 μg OX_2_ reduced βarrestin2 recruitment compared to cells without OX_2_ and when 0.10 μg OX_2_ was compared to 0.25 μg OX_2_ (Figure 3C). Cells that received 0.1% DMSO in PBS that were transfected with 0.25 μg OX_2_ displayed less βarrestin2 recruitment than cells transfected with 0.00, 0.10, or 0.50 μg OX_2_ (Figure 3C). Treatment with 0.1% DMSO in PBS also appears to have reduced βarrestin2 recruitment compared to untreated cells that received 0.00 or 0.10 μg OX_2_ (Figure 3C); as in Figure 2C. it is unknown whether this is biologically significant but is reported to show the vehicle did affect our observations under some conditions. The CRC for CP55,940 in CHO‐K1 CB_1_ βarrestin2 cells that were not transfected (same as in Figure 2D) did not reach a clear asymptote, and so the reported *E*_max_ is based on the observed result rather than the nonlinear regression (Figure 3D). Accordingly, the potency of CP55,940 in these experiments could not be accurately estimated (Figure 3D and Table 2). A statistically significant increase in the observed maximum response of CP55,940 was observed for all transfection groups (Figure 3D and Table 2). These data suggest that the presence of OX_2_ can reduce CB_1_‐mediated βarrestin2 recruitment in the absence of an agonist or facilitate CB_1_‐mediated βarrestin2 recruitment in the presence of an agonist. βarrestin2 recruitment was reduced as a function of increasing OX_2_ amounts, whereas CP55,940 treatment increased βarrestin2 recruitment as a function of increasing OX_2_ amounts.

**TABLE 2:** CB_1_‐dependent inhibition of cAMP and recruitment of βarrestin2 in CB_1_‐OX_2_ cells.

[OX_2_] (μg)	cAMP			βarrestin2		
	pEC_50_ (nM)	*E*_max_ (Fold)	Hill slope	pEC_50_ (nM)	*E*_max_ (Fold)^a^	Hill slope
0.00	8.8 ± 0.45 (1.5)	0.79 ± 0.03	−1.7 ± 3.6	< 5 (> 10 000)	17 ± 4.6	n.d.
0.10	9.4 ± 2.5 (1.3)	0.62 ± 0.07	−0.5 ± 0.66	< 5 (> 10 000)	33 ± 0.92*	n.d.
0.25	> 10 (< 0.1)	0.58 ± 0.06*	−0.39 ± 0.59	< 5 (> 10 000)	41 ± 1.5**	n.d.
0.50	> 10 (< 0.1)	0.43 ± 0.06***	−0.35 ± 0.45	< 5 (> 10 000)	39 ± 3.8**	n.d.

*Note:* Data were fit to a 4‐parameter non‐linear regression to estimate potency (pEC_50_) and efficacy (*E*_max_) values. Data are expressed as mean ± SEM. *n* = 4–8 independent experiments performed in triplicate. If potency could not be estimated (i.e., < 0.1 nM or > 10 000 nM), then *E*_max_ is reported as the mean of the greatest response observed. Statistical analyses were by one‐way ANOVA followed by Tukey's post hoc test. Data presented in the Table relate to Figure 3.

Abbreviation: n.d., not determined.

* *p* < 0.05.

** *p* < 0.01.

*** *p* < 0.001 relative to CP55,940.

^a^ *E*_max_ data represent the experimentally observed value because CRC did not reach a clearly defined maximum asymptote; for this reason EC_50_ is reported as > 10 000 nM.

### 3.4 Modulation of cAMP and βarrestin2 Recruitment in CB_2_ ‐OX_1_ Transfected Cells

**FIGURE 4:**
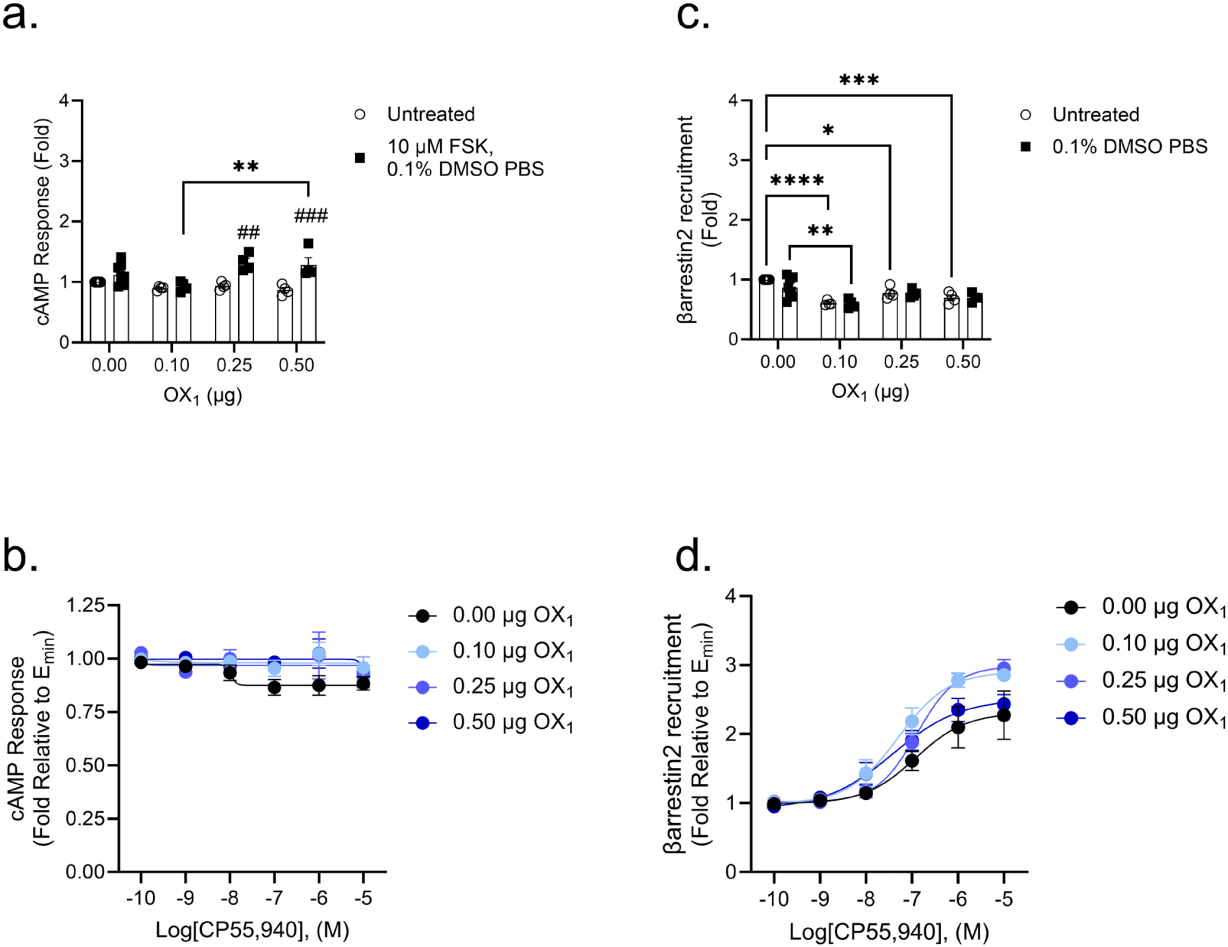
CB_2_‐dependent inhibition of cAMP and βarrestin2 recruitment in CB_2_‐OX_1_ cells. CHO cells stably expressing CB_2_ and transiently expressing OX_1_ were treated with 10 μM FSK and 0.1 nM–10 μM CP55,940 for 90 min to measure cAMP inhibition (A, B) or 0.1 nM–10 μM CP55,940 for 90 min to measure βarrestin2 recruitment (C, D). Data were fit to a 4‐parameter non‐linear regression and are represented as fold over vehicle. Data are mean ± SEM from *n* = 4–8 independent experiments performed in triplicate. *p < 0.05, **p < 0.01, ***p < 0.001, ****p < 0.0001 within treatment between groups; ^##^ *p* < 0.01, ^###^ *p* < 0.001 within groups between treatments as determined by two‐way ANOVA followed by Tukey's post hoc test.

No statistical difference in cAMP levels was detected between untreated cells receiving varying amounts of OX_1_ (Figure 4A). Ten micrometer FSK in 0.1% DMSO with PBS increased the cAMP response as expected compared to untreated cells, and this difference was statistically significant in cells receiving 0.25 and 0.50 μg OX_1_ compared to untreated cells (Figure 4A). Cells transfected with 0.10 μg OX_1_ that received 10 μM FSK in 0.1% DMSO and PBS saw a cAMP response that was lower compared to cells transfected with 0.50 μg OX_1_ (Figure 4A). As above, CRC data in Figure 4B,D were constrained for *E*_min_ = 1 to allow for comparisons of potency and efficacy following CP55,940 treatment. In cells that were not transfected with OX_2_, a concentration‐response for CP55,940 was observed with a potency of 10 nM if the Hill Slope was constrained to 1 (Figure 4B); however, the goodness of fit was relatively low (*R* ^2^ = 0.21) albeit with reasonable residual standard deviation (Sy.x = 0.092); therefore, these data should be interpreted with caution. Potency could not be determined for cells transfected with 0.10, 0.25, or 0.50 μg OX_1_, as no concentration‐response was clearly observed (Figure 4B and Table 3). The efficacy of CP55,940 decreased as the amount of OX_1_ increased, though this was only statistically significant for cells transfected with 0.10 μg OX_1_ (Figure 4B). These data suggest that OX_1_ prevents CB_2_‐dependent cAMP inhibition. Basal βarrestin2 recruitment was lower in untreated CB_2_‐expressing cells transfected with 0.10, 0.25, or 0.50 μg OX_1_ compared to non‐transfected cells; similarly, βarrestin2 recruitment was lower in CB_2_‐expressing cells transfected with 0.10 μg OX_1_ compared to non‐transfected cells that received 0.1% DMSO in PBS (Figure 4C). The potency of CP55,940 did not appear to follow a specific trend as a function of the amount of OX_1_ transfected (Table 3). However, the efficacy of CP55,940 did increase as the level of OX_1_ increased up to 0.25 μg OX_1_ (Figure 4D and Table 3). At 0.50 μg OX_1_, the efficacy of CP55,940 was not statistically different from that of non‐transfected cells (Table 3). Thus, our system may be oversaturated at plasmid levels above 0.25 μg OX_1_. These data suggest that OX_1_ enhances CP55,940‐dependent βarrestin2 recruitment while reducing βarrestin2 recruitment in the absence of a CB_2_ agonist.

**TABLE 3:** CB_2_‐dependent inhibition of cAMP and recruitment of βarrestin2 in CB_2_‐OX_1_ cells.

[OX_1_] (μg)	cAMP			βarrestin2		
	pEC_50_ (nM)	*E*_max_ (Fold)^b^	Hill slope	pEC_50_ (nM)	*E*_max_ (Fold)	Hill slope
0.00	8.0 ± 0.62 (10)^a^	0.87 ± 0.02^a^	−1^a^	6.9 ± 0.42 (120)	2.3 ± 0.27	0.79 ± 0.58
0.10	n.c.	0.96 ± 0.05*	n.c.	7.3 ± 0.17 (50)	2.9 ± 0.13*	0.80 ± 0.22
0.25	n.c	0.94 ± 0.07	n.c.	6.9 ± 0.10 (130)	3.0 ± 0.11*	1.0 ± 0.25
0.50	n.c	0.92 ± 0.04	n.c.	7.4 ± 0.29 (38)	2.5 ± 0.18	0.60 ± 0.25

*Note:* Data were fit to a 4‐parameter non‐linear regression to estimate potency (pEC_50_) and efficacy (*E*_max_) values. Data are expressed as mean ± SEM. *n* = 4–8 independent experiments performed in triplicate. If potency could not be estimated (i.e., < 0.1 nM), then *E*_max_ is reported as the mean of the greatest response observed. Statistical analyses were by one‐way ANOVA followed by Tukey's post hoc test. Data presented in the Table relate to Figure 4.

Abbreviation: n.c—not converged.

* *p* < 0.05 relative to CP55,940.

^a^ When fit to a 4‐parameter model, pEC_50_ and Hill Slope could not be accurately estimated, data in table are for a Hill Slope constrained to 1.

^b^ For data where no clear CRC was observed, *E*_max_ represents the lowest value observed.

### 3.5 Modulation of cAMP and βarrestin2 Recruitment in CB_2_ ‐OX_2_ Transfected Cells

**FIGURE 5:**
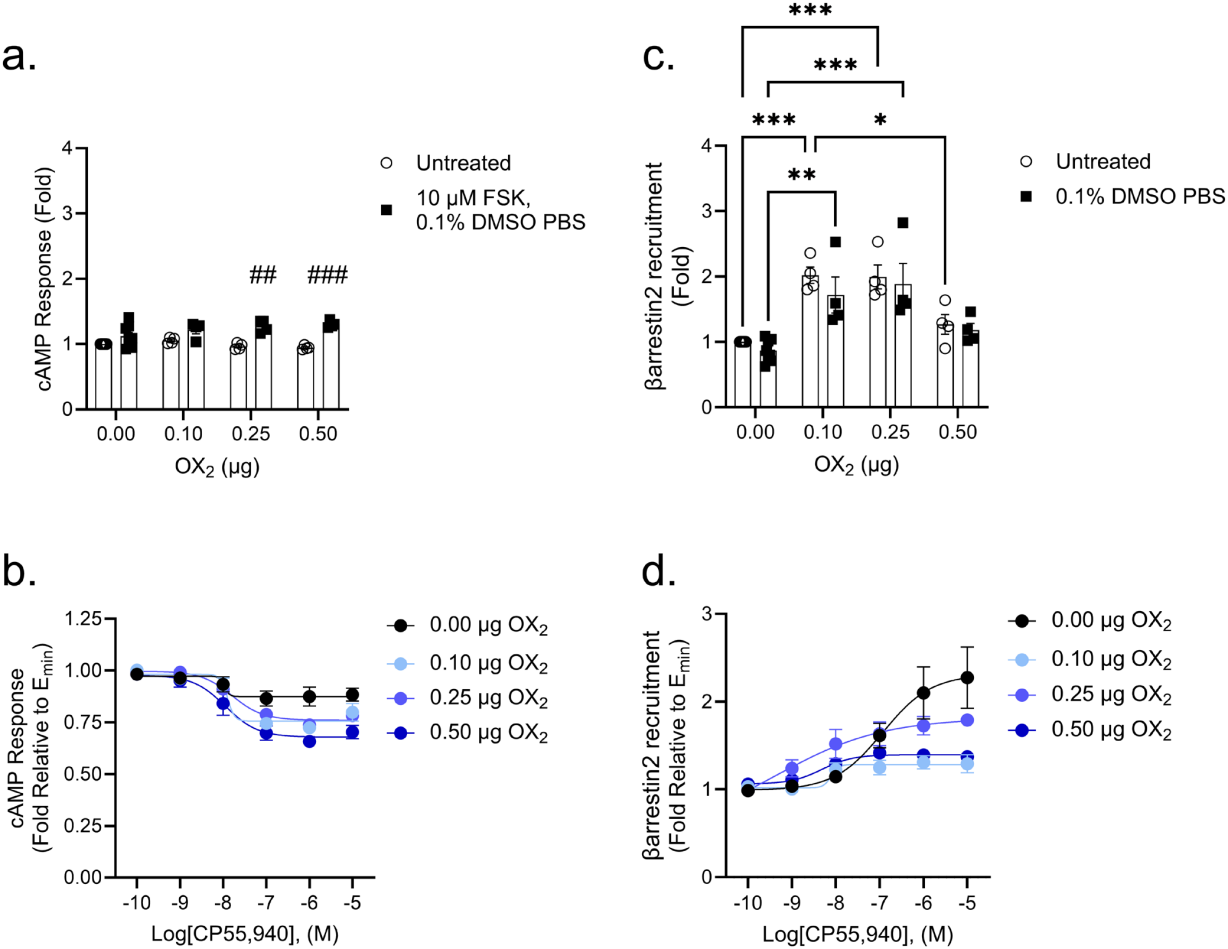
CB_2_‐dependent inhibition of cAMP and βarrestin2 recruitment in CB_2_‐OX_2_ cells. CHO cells stably expressing CB_2_ and transiently expressing OX_2_ were treated with 10 μM FSK and 0.1 nM—10 μM CP55,940 for 90 min to measure cAMP inhibition (A, B) or 0.1 nM—10 μM CP55,940 for 90 min to measure βarrestin2 recruitment (C, D). Data were fit to a 4‐parameter non‐linear regression and are represented as fold over vehicle. Data are mean ± SEM from *n* = 4–8 independent experiments performed in triplicate. *p < 0.05, **p < 0.01, ***p < 0.001 within treatment between groups; ^##^ *p* < 0.01, ^###^ *p* < 0.001 within groups between treatments, as determined by two‐way ANOVA followed by Tukey's *post hoc* test.

No statistical differences in cAMP response were observed among untreated cells (Figure 5A). Ten micrometer FSK (in 0.1% DMSO with PBS) produced an increase in cAMP response that was significant in cells that were transfected with 0.25 or 0.50 μg OX_2_ compared to untreated cells (Figure 5A). As elsewhere, CRC data in Figure 5B,D were constrained for *E*_min_ = 1 to allow for comparisons of potency and efficacy following CP55,940 treatment. Data for cells not expressing OX_2_ (i.e., 0.0 μg) or transfected with 0.10 μg OX_2_ could not be fit to a 4‐parameter model as the Hill slope was too variable (Figure 5B and Table 4). The CRC for cells that were not transfected with OX_2_ had a relatively low goodness of fit (*R* ^2^ = 0.21); and these data should be interpreted with that caveat in mind. CP55,940's efficacy significantly increased in all transfection groups in an OX_2_ amount‐dependent order (Figure 5B and Table 4). These data suggest that OX_2_ facilitates CB_2_‐dependent cAMP inhibition in CP55,940‐treated cells. In untreated cells, the presence of OX_2_ led to a significant increase in βarrestin2 recruitment in cells transfected with 0.10 and 0.25 μg OX_2_ but not in cells transfected with 0.50 μg OX_2_, which may suggest that our system is oversaturated at plasmid levels above 0.25 μg OX_2_ (Figure 5C). In cells that received 0.1% DMSO with PBS, a similar effect was seen, in which cells transfected with 0.10 and 0.25 μg OX_2_ exhibited significant increases in βarrestin2 recruitment (Figure 5C). Data for cells transfected with 0.10 μg OX_2_ could not be fit to a 4‐parameter model as the Hill slope was too variable (Table 4). CP55,940's potency was improved in the presence of 0.25 μg OX_2_, although its efficacy decreased in cells transfected with OX_2_, with only 0.10 μg being significant (Figure 5D and Table 4). Overall, OX_2_ enhanced basal CB_2_‐mediated βarrestin2 recruitment, but CP55,940's potency and efficacy at CB_2_ were generally reduced when OX_2_ was present.

**TABLE 4:** CB_2_‐dependent inhibition of cAMP and recruitment of βarrestin2 in CB_2_‐OX_2_ cells.

[OX_2_] (μg)	cAMP			βarrestin2		
	pEC_50_ (nM)	*E*_max_ (Fold)	Hill slope	pEC_50_ (nM)	*E*_max_ (Fold)	Hill slope
0.00	8.0 ± 0.62 (10)^a^	0.87 ± 0.02^a^	−1^a^	6.9 ± 0.42 (120)	2.3 ± 0.27	0.79 ± 0.58
0.10	7.8 ± 0.26 (17)^a^	0.75 ± 0.02**, ,^a^	−1^a^	8.3 ± 0.51 (5.0)^a^	1.3 ± 0.04*,^a^	1^a^
0.25	7.8 ± 0.17 (16)	0.76 ± 0.02**	−1.2 ± 0.56	9.3 ± 3.2 (0.45)*	1.8 ± 0.20	0.36 ± 0.45
0.50	7.9 ± 0.20 (11)	0.68 ± 0.03****	−1.2 ± 0.78	8.3 ± 0.32 (4.9)	1.4 ± 0.03	1.2 ± 0.86

*Note:* Data were fit to a 4‐parameter non‐linear regression to estimate potency (pEC_50_) and efficacy (*E*_max_) values. Data are expressed as mean ± SEM. *n* = 4–8 independent experiments performed in triplicate. Statistical analyses were by one‐way ANOVA followed by Tukey's post hoc test. Data presented in the Table relate to Figure 5.

* *p* < 0.05.

** *p* < 0.01.

*** *p* < 0.001.

**** *p* < 0.0001 relative to CP55,940.

^a^ When fit to a 4‐parameter model, pEC_50_ and Hill Slope could not be accurately estimated, data in table are for a Hill Slope constrained to 1.

## 4 Discussion

CB_1_ interacts with various serotonin, somatostatin, dopamine, opioid, adenosine, and angiotensin receptors, as well as the orphan receptor GPR55 [31, 32, 33, 34, 35, 36]. Some of these interactions are known to modulate receptor activity. For example, CB_1_ preferentially couples to Gα_s_ G proteins instead of Gα_i/o_ G proteins when co‐expressed with the dopamine D_2_ receptor [37]. Much less is known about CB_2_'s interactions with other GPCRs, though previous studies suggest CB_2_ interacts with GPR55, serotonin, and chemokine receptors [38, 39, 40, 41].

Data from numerous studies now suggest that crosstalk exists between the endocannabinoid and orexinergic systems reviewed in [12]. It is critical to clarify how these complex biological systems interact with one another, considering their shared anatomical distribution and what implications these interactions have on physiological processes regulated by these systems. Our results demonstrate that the introduction of OX_1_ or OX_2_ can alter the pharmacological properties (i.e., efficacy, potency) of the agonist CP55,940 at CB_1_ and CB_2_. For CB_1_, the presence of OX_1_ appeared to increase the efficacy of CP55,940‐dependent cAMP inhibition and conversely decrease βarrestin2 recruitment, with no clear effect on potency; whereas the presence of OX_2_ augmented the efficacy and potency of CP55,940‐dependent cAMP inhibition *and* the efficacy of βarrestin2 recruitment. For both CB_2_‐OX_1_ and CB_2_‐OX_2_ pairings, the presence of OX_1_ or OX_2_ appeared to decrease the efficacy of CP55,940‐dependent cAMP inhibition; whereas for βarrestin2 the CB_2_‐OX_1_ pairing was associated with greater βarrestin2 recruitment in the presence of CP55, 940 and the CB_2_‐OX_2_ pairing was associated with less βarrestin2 recruitment. These data suggest that the form of interaction between CB_2_‐OX_1_ or CB_2_‐OX_2_ and the Gα_i/o_ and βarrestin2 recruitment pathways differs from that of CB_1_ with OX_1_ or OX_2_ for that matter. Such differences in receptor interactivity warrant further examination in future studies to determine what effectors—whether at the receptor or downstream—differentiate each pairing and gain mechanistic insight into receptor crosstalk.

As with other studies exploring GPCR signaling and cross‐talk, a major limitation is that our results were obtained in a heterologous system [19, 42]. Considering that our in vitro model consisted of CHO‐K1 cells overexpressing human GPCRs, these results may not be reflective of the activity of these receptors when expressed in native tissues [43, 44]. In our experimental setup, the chosen vehicles (e.g., 0.1% DMSO in PBS) produced changes in cAMP response or βarrestin2 recruitment that were sometimes unexpectedly statistically significant (e.g., Figures 2C and 3C). Although the biological significance of these changes is unclear, we felt it important to include those data to showcase the real potential effects vehicle solvents can have on system measurements. Such systems are inherently variable, and here we display that variability as directly as possible. In addition, our study examined a single timepoint of receptor signaling, 90 min after CP55,940 treatment. This timepoint was optimized for the βarrestin2 assay and then matched in the cAMP inhibition assay. However, it is possible that receptor desensitization occurred during that time or that some signal was lost in the cAMP assay due to the actions of phosphodiesterases. Moreover, our study did not account for the potential activity of endogenous cannabinoids such as 2‐AG [20, 21]. Thus, we do not know if these effects detected in vitro would also be present in vivo. It will be necessary to establish that these receptor pairings are co‐expressed on the same cells or tissues in vivo [18]. Even if these GPCRs are co‐expressed and present in vivo, we will need to assess whether these receptors engage in crosstalk and/or heterodimerization [45]. Previous work from our research group using C57BL/6 mice found that OX_1_ antagonism increases CB_1_‐OX_1_, but not CB_1_‐OX_2_, colocalization in the ventral striatum, and that this colocalization is responsible for the observed increase in catalepsy in mice co‐treated with CP55,940 and the dual orexin receptor antagonist (DORA) TCS‐1102 [46].

While this study revealed that OX_1_ and OX_2_ can alter CB_1_‐mediated and CB_2_‐mediated signaling, our experiments used CHO‐K1 cells that were transiently transfected with OX_1_ or OX_2_. Transfection efficiency in these cells ranged from 19% to 75% but was higher than cells that were exposed to only Lipofectamine 3000 and not transfected with plasmid DNA (i.e., ‘mock' transfected). Increasing amounts of either receptor did produce a response different from mock‐transfected controls. Additionally, we did not establish the physical interaction of these receptors, which has been done by other groups in similar model systems already [12]. We aim to assess changes in receptor trafficking, co‐localization, and expression via immunohistochemical microscopy and western blotting with in vitro and ex vivo model systems in future studies. It is possible that the observed effect of orexin receptors on cannabinoid receptor signaling is a result of a functional interaction and not evidence of receptor dimerization per se [22]. Bioluminescence resonance energy transfer (BRET) and/or fluorescence resonance energy transfer (FRET) techniques could also be used to investigate physical interactions between these receptors [33]. However, such experiments would only establish whether heterodimer formation between cannabinoid and orexin receptors is possible and not if such complexes alter signal transduction at the plasma membrane [23, 31].

It is important to determine the specific physiological roles of cannabinoid‐orexin receptor crosstalk and this will require tools which probe those interactions (e.g., heterodimer‐specific agonists and antagonists) [33]. The development of bitopic or bivalent ligands targeting cannabinoid‐orexin heterodimers will be of great use to study these interactions in vivo and to determine their significance in physiological or pathological conditions [7, 47]. Such ligands consist of two pharmacophores joined together by a linker molecule allowing them to simultaneously interact with both receptors of the heterodimer [6, 42]. A series of bivalent ligands targeting CB_1_ and OX_1_ has been previously developed using SR141716 and ACT‐078573 pharmacophores. However, while all ligands in this series were potent at CB_1_, most had poor activity at OX_1_, demonstrating the need for bivalent ligands with improved pharmacological properties at both receptors of the heterodimer [7].

In conclusion, our study revealed that orexin receptors affect agonist‐dependent signaling at the cannabinoid receptors. Orexin receptors generally enhanced cannabinoid receptor‐mediated cAMP inhibition while they may increase or decrease cannabinoid receptor‐mediated βarrestin2 recruitment. The effects on cannabinoid receptor signaling by the presence of orexin receptors observed here require further evaluation regarding receptor localization and expression both in vitro and in vivo.

## Author Contributions

K.A.M. designed and executed experiments, analyzed data, and contributed to the writing and editing of the manuscript. R.B.L. analyzed data and contributed to the writing and editing of the manuscript.

## Conflicts of Interest

R.B.L. served on the Scientific Advisory Board for Shackleford Pharma Inc. and has consulted in medico‐legal cases concerning cannabis and cannabinoids. None of these entities had any actual or perceived input into the design or output of this study. K.A.M. declares that the research was conducted in the absence of any commercial or financial relationships that could be construed as a potential conflicts of interest.

## Supporting information


Data S1.

## Data Availability

The authors declare that all the data supporting the findings of this study are available within the paper and its Supplemental Data. Raw data used for this study are available at the Dryad repository (DOI: 10.5061/dryad.0cfxpnwcq).
